# From graft survival to life: redefining success after pediatric liver transplantation

**DOI:** 10.3389/ti.2026.16481

**Published:** 2026-06-24

**Authors:** Geraldine Dahlqvist, Clara Gautier, Xavier Stephenne

**Affiliations:** 1 Cliniques universitaires Saint-Luc, Service d’hépatogastroentérologie, Brussels, Belgium; 2 Cliniques universitaires Saint-Luc, Service de gastro-pédiatrie, Brussels, Belgium

**Keywords:** long-term outcome, meaningful survival, transition, pediatric liver transplantation, adherence

## Abstract

Over four decades, pediatric liver transplantation represents a major medical achievement. Graft function and biochemical stability alone no longer define success. Recent data show that only a minority of long-term survivors meet composite criteria for “meaningful” survival, which includes psychological wellbeing, social integration, and treatment adherence. Growing up with a transplant shapes identity, autonomy, and life trajectory. Although many recipients achieve education, employment, and parenthood, patient-reported outcomes reveal persistent psychological distress, reduced health-related quality of life, and ongoing vulnerability. Mental health symptoms, limited health literacy, and gaps in disease understanding may compromise adherence and long-term outcomes. Substance use, while not necessarily more prevalent than in the general population, carries disproportionate risks in this fragile group and underscores the need for early, structured prevention. Transition from pediatric to adult care represents a critical pivotal and formative period, marked by increased risk of nonadherence and graft complications or graft loss, but also a unique opportunity to strengthen autonomy, psychoeducation, and self-management skills. We argue for a paradigm shift toward lifelong, multidisciplinary follow-up integrating psychological screening, structured transition pathways, and sustained health education. Pediatric transplantation has created long-term survivors. The next challenge is to ensure they become informed, autonomous, and psychologically supported adults.

## Introduction – a shift in paradigm

Pediatric liver transplantation (LT) has been performed for over four decades and remains a complex and demanding procedure. It represents a major medical achievement, whose success cannot be assessed solely by postoperative outcomes, graft function, biochemical parameters, or patient survival. Increasingly, attention has shifted toward broader measures of outcome, including the patient’s ability to lead a meaningful and “normal” life [1]. Several studies evaluating long-term outcomes after pediatric liver transplantation have attempted to define an “ideal outcome profile.” This profile includes survival with the first graft, stable immunosuppression on monotherapy, normal growth and development, and the absence of significant immunosuppression-related complications [2, 3]. Strikingly, a 10-year follow-up study revealed that only 32% of these patients achieved an “ideal profile” [4, 5]. In line with this broader perspective, a recent long-term analysis conducted at a median follow-up of 20 years introduced the notion of “meaningful survival” as a more comprehensive benchmark. Beyond preserved liver function, renal function, and body mass index, this definition incorporated the absence of diagnosed mental health disorders, active engagement in education or employment, and treatment adherence exceeding 80%. Strikingly, only 26% of pediatric liver transplant recipients fulfilled these combined criteria. Considering these results, the focus must evolve from graft survival alone to a larger concept of survival [3]. Indeed, psychological evolution and social integration have received increasing attention in research, reflecting a shift toward more comprehensive models of post-transplant evaluation. The traditional assessment of LT success through medical and biological parameters often fail to capture the full complexity of the post-transplant experience [3].

The central argument of this viewpoint paper is to propose the concept of “meaningful survival” as a clinical standard. We want to move beyond isolated markers of graft function toward a structured, multidimensional framework, integrating psychological wellbeing, autonomy, health literacy and social integration. This should inform the organization of the follow-up care, help designing multi and interdisciplinary transition programs, and allocate adequate resources in pediatric liver transplantation.

We have recently published a study in Pediatric Transplantation, aiming to illustrate this turning point and to highlight what remains insufficiently explored. To this aim, we evaluated psychosocial, behavioral, and lifestyle outcomes of fifty adults who received a liver transplant during childhood, using validated tools and a fully anonymous design. Our findings revealed persistent psychological distress and limited disease understanding well into adulthood, even among medically stable individuals [6].

In this viewpoint article we will discuss the long-term psychosocial consequences of being transplanted in childhood and highlight the data that are missing to improve patient care beyond disease specific issues. We will also propose a practical framework for integrating these dimensions into routine practice.

## Growing up with a transplant: a unique life trajectory

Liver transplantation during childhood represents a formative life event that shapes personal identity. The acute urgency of surgery and survival gradually gives way to lifelong medical surveillance extending far beyond graft function, including monitoring for immunosuppression-related complications [7]. Childhood is marked by medicalization, dependence on care, and a distinct relationship to the body and illness.

In qualitative interviews with 27 long-term survivors of pediatric LT, Lawton et al. described how adult recipients navigate between narratives of normality and difference, as their embodied transplant experience continues to shape identity construction [8]. These tensions vary according to context: within the family or hospital environment, where transplantation is framed as a miracle restoring normality, and within broader society, namely at school, where social identity is renegotiated and sometimes questioned.

Even with excellent medical outcomes, transplantation remains a lifelong vulnerability. These grown-up children are considered as “survivors”. They live with a chronic latent stress with uncertainties regarding their future, struggling with the fear of potential graft loss or retransplantation in the future and adapting their choice and lifestyle to their condition.

## A “normal” adult life… but not entirely

Many individuals transplanted during childhood reach key milestones of “normal” adult life and social integration, namely education, employment, stable relationships, and parenthood. In a British cohort of 143 survivors with more than 15 years of follow-up (median 19.5 years), 92% had completed or were pursuing education, 46.3% of those transferred to adult care were employed, and 67.5% were in a relationship [9]. Overall, these data suggest that most pediatric liver transplant recipients integrate successfully into adult social and professional life, often at levels comparable to the general population.

Parenthood is feasible and appears to be associated with predominantly favorable maternal and neonatal outcomes, although available series remain small and underscore the need for more systematic, long-term data. At approximately 20 years of follow-up, 17.5% of patients in the study by Cussa et al. and 10.6% in the study by Ruth et al. had at least one child [9, 10].

Yet, this encouraging picture must be balanced against more nuanced patient-reported outcomes. Pediatric liver transplant recipients consistently report lower health-related quality of life (HRQOL) compared with healthy peers, with overall scores comparable to those observed in other chronic pediatric conditions. In adulthood, some studies describe HRQOL similar to that of healthy controls, but also highlight that more than half of patients report anxiety, negative thoughts, perceived limitations in autonomy, and feelings of loneliness [11]. Two decades after transplantation, lower physical HRQOL and reduced health utility scores have been documented [12]. Importantly, higher HRQOL and health utility scores are associated with employment and higher educational attainment, whereas a more complex medical history and repeated hospitalizations during childhood negatively impact long-term wellbeing. Patients who achieve operational tolerance and discontinue immunosuppressive therapy tend to report better HRQOL outcomes [12].

These findings must be interpreted cautiously. Most available data derive from survey-based studies. Lost to follow-up may bias estimates of long-term outcomes and risk factors for poor outcomes [13]. Non-responders frequently represent more vulnerable individuals. In one study, the 65 nonparticipants had greater psychological difficulties and lower educational levels than participants [11]. Thus, the long-term psychosocial burden may be underestimated.

In addition, social functioning does not preclude deep psychological vulnerability.

## Mental health: the blind spot of pediatric transplantation

Psychological symptoms such as anxiety, depression, and post-traumatic stress are well known to influence post-transplant outcomes. Pediatric liver transplant recipients exhibit substantial rates of anxiety and depressive symptoms, and our recently published study, based on validated anonymous surveys, which included 50 adult patients transplanted before 18 years of age, highlighted this often hidden burden [6]. Using validated questionnaires for the screening of anxiety and depression, as well as screening of alcohol use disorders, we found that clinically relevant anxiety and depressive symptoms were reported by 49% and 33% of respondents, respectively. Despite these challenges, 76% of participants were professionally active, underscoring the limited predictive value of social integration as a proxy for psychological wellbeing. A well-functioning graft cannot be assimilated with a patient who is well. A more holistic view is needed.

Data from adult transplant populations consistently demonstrate associations between post-traumatic symptoms, anxiety, depression, and poorer graft-related outcomes, particularly through impaired adherence and altered illness perception [14, 15]. However, some of these considerations may not fully apply to individuals transplanted in early childhood, many of whom have no autobiographical memory of the transplant itself. Their psychological trajectory differs fundamentally from that of patients transplanted in adolescence or adulthood.

This heterogeneity is rarely accounted for in current studies. Age at transplantation likely plays a critical role: children transplanted before the age of two, those transplanted between early childhood and preadolescence, and those transplanted during adolescence may experience and internalize the transplant differently. Children transplanted at a younger age (especially before age two) are more likely to have neurocognitive and developmental challenges, including deficits in motor skills, receptive language, and cognitive functioning, which can persist into adolescence and adulthood [16]. Adolescents are particularly vulnerable to psychological distress due to the intersection of transplant-related stressors and normal developmental tasks, including peer integration and self-management of health [17]. The psychological impact of acute liver failure leading to urgent transplantation may also differ from that of chronic disease. Furthermore, parental socioeconomic status, educational background, and family dynamics may significantly shape long-term psychological adjustment [18, 19].

However, there is insufficient granularity in the literature regarding the impact of demographic, socioeconomic, and transplant-related variables (e.g., age at transplant, indication, immunosuppressive regimen, graft complications) on psychological distress in this population. Existing studies often fail to stratify outcomes according to these factors or lack sufficient power to detect meaningful subgroup differences. In addition, standardized and validated tools for assessing psychological distress are inconsistently applied, and outcome measures remain heterogeneous, limiting comparability across studies.

What remains certain is that a comprehensive, individualized approach to care, integrating psychological considerations alongside surgical and immunological management, is essential for the overall success of transplantation. In this context, we should consider psychological wellbeing as a multidimensional construct encompassing psychological distress (anxiety, depression, post-traumatic symptoms); illness perception and healthcare literacy; coping strategies and resilience; social function and finally autonomous self-management of health. Psychological wellbeing strongly influences medication adherence, illness understanding, risk perception, social integration, and ultimately overall quality of life. Some young adults demonstrate a limited understanding of their disease or, conversely, a trivialization of medical risks, both of which may compromise long-term outcomes.

Systematic monitoring of emerging psychiatric symptoms would allow earlier identification of vulnerable individuals and facilitate timely intervention, thereby mitigating the long-term impact of psychological distress on quality of life and graft outcomes. In routine practice, we should use systematic, validated, brief screening tools alongside structured assessment of adherence and health literacy.

## The importance of prevention: addictions and risk behaviors

Adolescents and young adults who underwent liver transplantation during childhood are at risk for substance use and other high-risk behaviors, including alcohol, nicotine, cannabis use, and medication nonadherence [11, 20, 21]. Although data specific to transplant recipients remain limited, available studies suggest that overall rates of substance use are similar or slightly lower than those observed in the general population [20]. However, the consequences may be considerably more severe in this medically vulnerable group.

Substance use not only increases the risk of nonadherence, a well-established determinant of graft failure, but may also directly damage the graft through direct organ toxicity and interactions with immunosuppressive regimens. Alcohol use among individuals transplanted during childhood is reported in 28%–43% of recipients [22]. Identified risk factor for harmful drinking in liver transplant recipients are male gender, the patients transplanted before 18 years of age, and increasing time since transplantation. Thus, childhood transplantation itself may represent a risk factor for later problematic alcohol use. In our cohort, 8% of participants showed problematic alcohol use as assessed by the (Alcohol Use DIsorders Test) AUDIT and, importantly 24% reported never having received medical counseling about alcohol risks after transplantation. Interestingly, substance use was not related to anxiety and depressive symptoms in our cohort, suggesting that risk behaviors may be driven less by psychiatric distress *per se* than by gaps in continuous education and risk awareness [6, 17].

We should emphasize the need for screening through validated tools such as the AUDIT In addition, we need to improve education of the patients and caregivers regarding substance-use-related risks after transplantation.

Substance use in this population should therefore not be trivialized, even when prevalence appears modest. Early initiation and regular consumption may have disproportionate consequences for graft health and long-term survival. Prevention must begin early (between 9 and 12 years old) and should evolve progressively [20]. Discussions should occur both in the presence of parents and during confidential consultations without them, as confidentiality is essential to facilitate honest disclosure. Anticipatory guidance can help normalize screening and reduce anxiety for both patients and families.

Management should be multidisciplinary, involving transplant physicians, psychologists, and social workers. Identification of substance use should prompt supportive, nonjudgmental intervention focused on education, motivational interviewing, and reinforcement of positive behaviors rather than moralization [23]. The goal is not only to prevent toxic injury to the graft but also to strengthen adherence, autonomy, and long-term health literacy.

Prevention strategies must extend beyond adolescence and continue into adulthood. If transplantation success is to be redefined beyond graft survival, then the monitoring and prevention of risk behaviors must become an integral component of long-term transplant care.

## Transition: a critical and formative moment

If meaningful survival is to extend beyond graft function alone, transition represents a pivotal developmental window during which the foundations of adult life are consolidated. Transition is not merely the administrative transfer from pediatric to adult services. It is a structured, multidisciplinary process that begins in adolescence and extends into early adulthood, aiming to progressively shift from a child- and family-centered model of care to a patient-centered model grounded in autonomy, self-management, and shared responsibility [24, 25].

Nevertheless, this period is frequently experienced as destabilizing. The loss of long-standing pediatric relationships, increased expectations of autonomy, and concurrent developmental challenges make transition a vulnerable phase. It is precisely during this time that nonadherence peaks and the risk of graft-related complications increase.

Drug nonadherence during transition has been reported in 24.8% of young adult recipients and is associated with significantly higher rates of donor-specific antibodies, elevated transaminases, and chronic rejection [2]. These data highlight that adherence is not merely a behavioral issue but a determinant of long-term graft survival. In parallel, psychological vulnerability remains prevalent. In one study of 187 young people, 17.7% screened positive for anxiety or depression [26]. In our recent study, a substantial proportion of participants reported moderate to severe depressive and anxiety symptoms, highlighting the close interconnection between psychological distress and self-management [6].

A well-structured transition process therefore represents a unique opportunity, not only to transfer care, but to reassess psychological wellbeing, reinforce disease knowledge, address risk behaviors, and discuss genetics, sexuality, parenthood, and life planning. Most importantly, it is a space to build autonomy in a progressive and supported manner [24, 27].

Health literacy is central to this process [27]. Although sufficient data are lacking regarding its impact on transition outcomes in pediatric liver transplantation, limited health literacy has been identified in 57% of recipients and 47% of caregivers in a recent study. In our cohort, only 31% of participants could correctly explain the pathophysiology of their liver disease, and 36% were unaware of the genetic nature and potential heritability of their condition. These figures are ethically concerning. Patients who are unaware of the genetic implications of their liver disease may be unable to make fully informed reproductive decisions or to alert at-risk family members. These data highlight the need to revisit not only medication management, but also genetic counseling and reproductive guidance as part of structured transition programs [6].

Numerous guidelines describe the components of an optimal transition program, and the needs of this period are increasingly well defined [28, 29]. However, real-world implementation often falls short, constrained by limited manpower, time, and institutional resources. Institutional and public health support are therefore indispensable to ensure that transition programs are adequately structured and resourced [25].

Based on these considerations, we propose that long-term follow-up and transition care should include a structured set of minimum components (Table 1) We should build structured interdisciplinary, coordinated programs, adapted to patient’s developmental stage and local resources. This framework is intended to serve as a practical guide for transplant teams and hepatologists in their routine practice.

**TABLE 1:** Proposed minimum components of long-term follow-up and transition pathway in pediatric liver transplant recipients.

Minimum components	Recommended evaluation	Suggested tools/Approach
*Adherence assessment*	Structured review of medication adherence; monitoring of immunosuppressant levels variability	Drug trough levels Drug levels variability
*Health literacy*	Structured assessment of disease understanding including if needed genetic implications, and treatment rationale	Structured disease knowledge checklist
*Genetic and reproductive counseling*	Provide information about genetic nature and heritability of the underlying liver disease; discuss pre conceptional councelling	Dedicated consultation with geneticist if needed
*Sexuality and reproductive health*	Discuss contraception, pregnancy planning, risk for drug teratogenicity (mycophenolate mofetil)	Dedicated consultation with gynecologist
*Risk behavior*	Education about risk for alcohol, tobacco, cannabis and other substance use (begin between 9–12 years) Regular confidential screening for several drug abuse	Motivational interviewing Confidential consultation AUDIT (alcohol)
*Social integration*	Assess educational and professional status, relationships, quality of life, referral to social worker when needed	Structured interview; SF-36 [12, 30] or pediatric quality of life 4.0 generic core scale for quality of life [31] evaluation
*Mental health*	Screening for anxiety, depression	STAI-Trait, BDI-SF [6]
*Transition readiness*	Assess autonomy and self-management skills. Structured transition program, structured document	Assessment TRAQ [25, 32]

An effective and well-supported transition should be viewed as a long-term investment in public health. By strengthening adherence, improving health literacy, and addressing psychological vulnerability, such programs may reduce psychiatric morbidity, graft loss, medical complications, and risk behaviors, including substance misuse. Importantly, transition frameworks developed in pediatric liver transplantation may serve as models for other rare and complex chronic diseases, with adaptation to disease-specific needs and vice-versa.

## Perspectives – toward a truly successful transplantation

Coming to the end of this viewpoint, it becomes clear that meaningful survival in pediatric liver transplantation cannot be reduced to long-term graft function or biochemical stability. The population we are now caring for is no longer defined by early postoperative survival, but by decades of life lived with a transplant. This reality calls for a shift in responsibility.

A better understanding and systematic identification of long-term needs and vulnerabilities should guide the next phase of transplant medicine. Lifelong screening for psychological distress and emerging psychiatric symptoms must become part of routine follow-up rather than an optional adjunct. These dimensions are not secondary outcomes; they are determinants of adherence, autonomy, and ultimately graft survival.

Transition represents a critical window. Although transition guidelines are well described, implementation remains uneven and often limited by resources. Institutional support is essential. Investment in structured transition and integrated psychological care should be viewed as a long-term public health strategy, capable of reducing psychiatric morbidity, nonadherence, graft loss, and risk behaviors.

Structured psychoeducation should continue into adult care. Disease understanding, genetic implications, reproductive counseling, and risk behavior education must be revisited across developmental stages.

Importantly, these individuals should be recognized as adults living with a transplant, not as “former pediatric patients.” Their care must reflect adult life projects while acknowledging the lasting imprint of early transplantation.

Pediatric transplantation has created long-term survivors. Our responsibility now is not only to ensure graft survival, but to create informed, autonomous, and psychologically supported adults.

## Data Availability

The original contributions presented in the study are included in the article/supplementary material, further inquiries can be directed to the corresponding author.
